# Management of recurrent mechanical prosthetic tricuspid valve thrombosis in the perioperative period of noncardiac surgery: a case report

**DOI:** 10.1186/1752-1947-6-150

**Published:** 2012-06-12

**Authors:** Amena Hussain, Tudor Vagaonescu

**Affiliations:** 1 Department of Cardiovascular Diseases and Hypertension, Robert Wood Johnson University Hospital, 5th floor MEB, 1 RWJ Place, New Brunswick, NJ, 08901, USA

## Abstract

**Introduction:**

Mechanical valves in the tricuspid position may be prone to thrombosis with even brief lapses in anticoagulation. The management of patients with a history of recurrent mechanical tricuspid valve thrombosis who require noncardiac surgery is complex.

**Case presentation:**

A 43-year-old Pakistani woman with a mechanical tricuspid valve and a history of multiple episodes of mechanical valve thrombosis presented for noncardiac surgery. After her surgery she was found to have another episode of valve thrombosis and underwent a redo tricuspid valve replacement.

**Conclusion:**

This case brings up the important dilemma that exists when patients with a mechanical tricuspid valve and a history of recurrent valve thrombosis require noncardiac surgery.

## Introduction

Tricuspid valve (TV) replacement is not a common operation and low flows on the right side of the heart increase the risk of thrombosis. Thrombolytics have had good results and are the initial treatment for a thrombosed mechanical TV [1-3]. In patients who are in a recent postoperative state, however, thrombolytics are not an option.

## Case presentation

A 43-year-old Pakistani woman presented for gynecologic surgery for an ovarian mass. Seventeen years prior, she had an emergency TV replacement with a St Jude bileaflet tilting disc prosthetic valve for bacterial endocarditis of her TV. The reasons for choosing a mechanical valve at that time are not known. Anticoagulation with warfarin without an antiplatelet agent was maintained and managed largely by our patient along with her primary care doctor. After her TV replacement she had three episodes of TV thrombosis, for which she received thrombolytics. Two of these episodes occurred during the discontinuation of warfarin and initiation of heparin or low molecular weight heparin for pregnancy, necessitating the termination of her pregnancies. Her international normalized ratio on admission was at a therapeutic level of 2.5; heparin was initiated with a goal prothrombin time of 80 seconds and warfarin discontinued for the gynecologic surgery. A preoperative echocardiogram showed a mean gradient across the TV of 5 mmHg.

**Figure 1:**
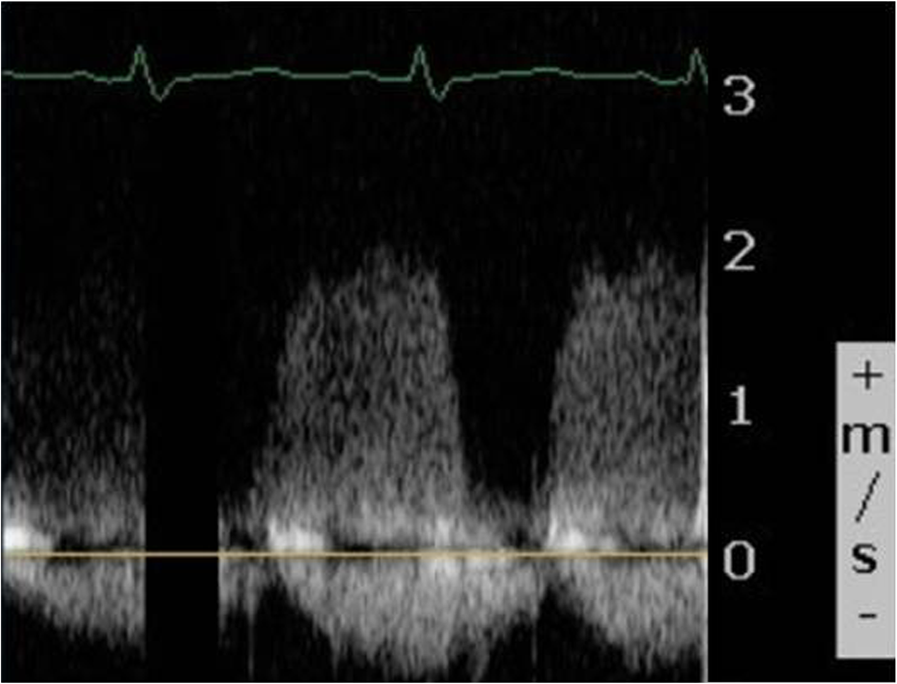
Continuous wave Doppler across the tricuspid valve showing increased inflow velocities.

**Figure 2:**
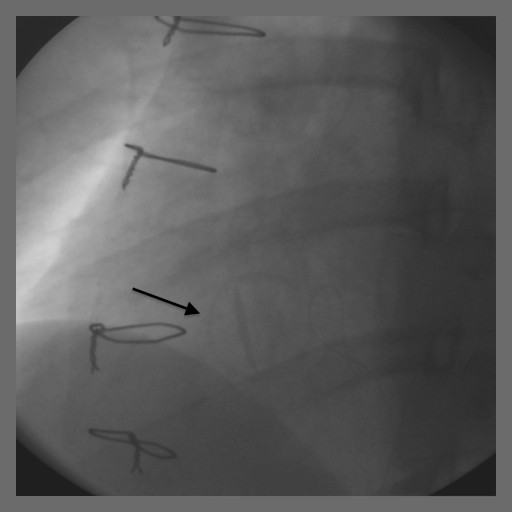
Fluoroscopy of the St Jude tilting disc tricuspid valve in different phases of the cardiac cycle, showing the valve to be stuck in the partially open position (systole).

**Figure 3:**
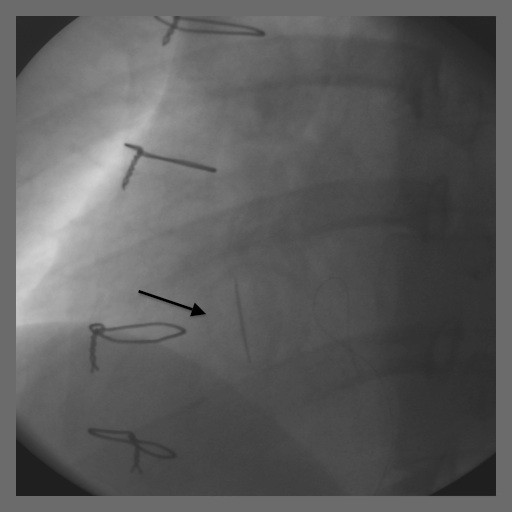
Fluoroscopy of the St Jude tilting disc tricuspid valve in different phases of the cardiac cycle showing the valve to be stuck in the partially open position (diastole).

Our patient underwent an exploratory laparotomy, oophorectomy, removal of an ovarian mass and a cystotomy repair. The surgery was four hours long and she had 400 cm^3^ of blood loss. Heparin was off for a total of 13 hours. On postoperative day one, our patient complained that she could not hear the click of her mechanical valve. She was hemodynamically stable with a heart rate of 105 beats per minute. Doppler echocardiography showed a mean TV gradient of 18 mmHg (Figure 1). A transesophageal echo showed the St Jude valve to be stuck in the open position. This was confirmed by fluoroscopy (Figure 2 and 3).

**Figure 4:**
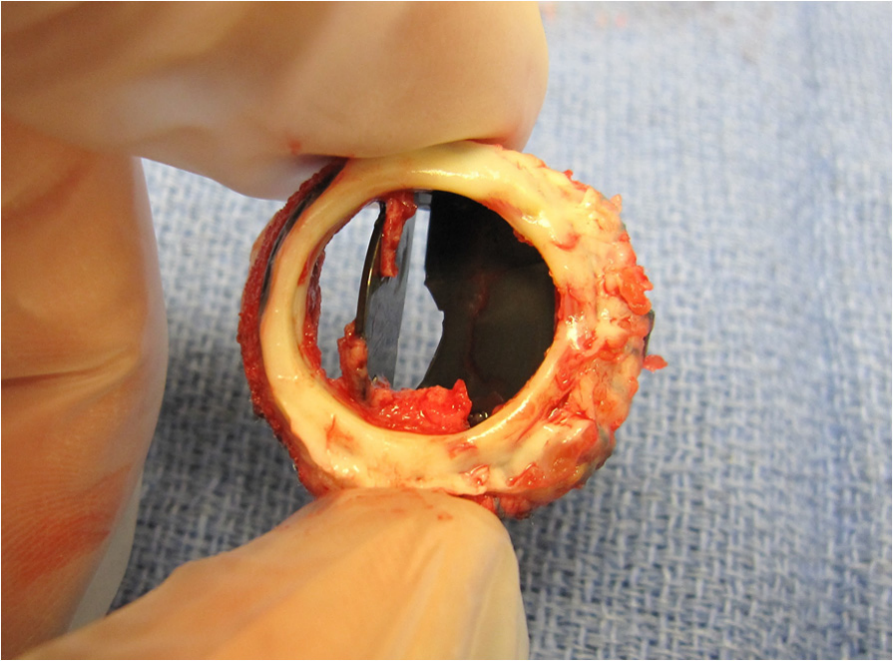
Explanted prosthesis showing fibrin and pannus involving both discs.

Thrombolytics were not an option as she was in a recent postoperative state. She was maintained under close observation and serial echocardiograms showed that the gradients across her TV remained stable. Limited blood testing did not show any disorders in blood coagulation. Ten days after her initial surgery, when it was deemed safe to be placed on bypass, her St Jude TV was removed and a bioprosthetic valve was placed in the TV position. The explanted prosthesis showed fibrin and pannus that involved both discs (Figure 4). Our patient tolerated the procedure well and went home in stable condition.

## Discussion

This case illustrates the dilemma associated with the need for noncardiac surgery in a patient who has had recurrent episodes of mechanical TV thrombosis.

TV replacement is not a common operation. Indications include severe primary tricuspid regurgitation with symptoms. The Society for Thoracic Surgeons national database reveals that approximately 5,800 TV surgeries, both repair and replacements, were performed in North America as compared to approximately 211,000 mitral valve surgeries [4]. In most cases, TV repair with annuloplasty is considered the procedure of choice however this is not always possible due to severe disease. TV replacements are done at approximately one-eighth the frequency as repairs [5]. The low velocity of blood across the right-sided heart valve makes it more prone to thrombosis, with the risk being up to 20%, especially in the setting of poor anticoagulation [6,7]. The 20% incidence of valve thrombosis cited by Thorburn *et al*. in monoleaflet models (mainly Bjork-Shiley) corresponds to an incidence of 4% per patient-year. [7] This has improved with the use of bileaflet valves to an incidence approaching 1% [8,9]. Most studies do not show a clear superiority of bioprosthetic versus St Jude mechanical valves though experience is limited [10-12]. A large meta-analysis in 2007 concluded that there were no major differences with the insertion of a mechanical versus a biological TV [13]. Some studies do show superiority of the bioprosthetic valves and these authors conclude that it is their valve substitution of choice, though neither choice is clearly a gold standard [14,15].

Thrombolytics have had good results and are the initial treatment for a thrombosed mechanical TV [1-3]. In patients who are in a recent postoperative state, thrombolytics are not an option due to the high risk of bleeding. Our patient underwent TV replacement with a bioprosthetic valve after her noncardiac surgery when a thrombus was suspected. Cardiac surgery was cautiously delayed for a period during which she had close hemodynamic monitoring to reduce the risk of bleeding from her recent surgical site.

## Conclusion

The management of patients with a history of recurrent mechanical TV thrombosis who require noncardiac surgery is complex. The elective replacement of a mechanical TV with a bioprosthetic valve before noncardiac surgery to lower the thrombotic risk would be considered too radical. Thrombolytics are the initial treatment for thrombosed mechanical valves but would be more dangerous in the postoperative state [1-3]. Reoperation of prosthetic valves has been shown to be low risk overall and new generations of bioprotheses have been shown to last longer [16]. The situation is unusual in that there are not many mechanical TVs placed, yet less so given the significant risk of thrombosis of the right-sided heart valve. Having to stop anticoagulation therapy in patients with mechanical valves for a period of time is common and pertinent to primary care physicians and surgeons, which makes education regarding valve choices and management of thrombotic complications important.

## Consent

Written informed consent was obtained from the patient for publication of this case report and any accompanying images. A copy of the written consent is available for review by the Editor-in-Chief of this journal.

## Competing interests

The authors declare that they have no competing interests.

## Authors’ contributions

AH wrote up the case report and discussion. TV revised the manuscript. Both authors were closely involved with the care of the patient and the acquisition of the images. Both authors read and approved the final manuscript.
